# Quantifying the contribution of individual variation in timing to delay-discounting

**DOI:** 10.1038/s41598-021-97496-w

**Published:** 2021-09-15

**Authors:** Evgeniya Lukinova, Jeffrey C. Erlich

**Affiliations:** 1 NYU-ECNU Institute of Brain and Cognitive Science at NYU Shanghai, Shanghai, 200062 China; 2 NYU Shanghai, Shanghai, 200122 China; 3 Shanghai Key Laboratory of Brain Functional Genomics (Ministry of Education), East China Normal University, Shanghai, 200062 China

**Keywords:** Psychology, Human behaviour

## Abstract

Delay-discounting studies in neuroscience, psychology, and economics have been mostly focused on concepts of self-control, reward evaluation, and discounting. Another important relationship to consider is the link between *intertemporal choice* and *time perception*. We presented 50 college students with timing tasks on the range of seconds to minutes and intertemporal-choice tasks on both the time-scale of seconds and of days. We hypothesized that individual differences in time perception would influence decisions about short experienced delays but not long delays. While we found some evidence that individual differences in *internal clock speed* account for some unexplained variance between choices across time-horizons, overall our findings suggest a nominal contribution of the altered sense of time in intertemporal choice.

## Introduction

The nature of time, and our relationship to time, continues to be a great puzzle to philosophers, physicists, psychologists and neuroscientists^1,2^. One consequence of dealing with time that has received great attention from economists, psychologists and neuroscientists is *delay discounting* (also named intertemporal choice in the literature): the tendency to consider a reward in the future to be worth less than an immediate reward. Many decisions we face involve this trade-off: choosing between an outcome (usually larger) to be received later and an outcome (usually smaller) to be received sooner. The variability in human’s delay discounting, starting from early childhood, is correlated with many measures of success later in life^3,4^. In a recent study, we compared subjects’ delay discounting for offers in seconds (the seconds task) with those in days (the days task) to investigate whether the two time-horizons engaged similar cognitive processes^5^. We found that the choices in the days task explained around 40% of the variance of choices in the seconds task: a substantial portion, but also leaving the majority of variance unexplained. Given that a substantial ($\sim 60\%$) fraction of the variance in one task was not explained by the other task, we considered differences in the tasks that could contribute to the unshared variance. One important difference was that all delays in the seconds task were experienced during the session (e.g., if a subject chose 10 coins in 30 s, then an individual would have to sit and wait for 30 s to receive the coins before proceeding to the next trial). In contrast, in the days task each choice was recorded and at the end of the session one trial was implemented. If the subject chose a delayed reward on that trial, they went about their lives and received an electronic payment at the appropriate time. Since subjects were more likely to pay attention to the duration of the delay in the seconds tasks, we hypothesized that individual differences in time perception seem more likely to influence choices in the seconds task than in the days task.

*Time perception* or the sense of the rate of time can vary from one person to the next^6,7^. In other words, after standing in line for 1 min, one person might report that 30 s had passed, while another might report that 2 min had passed. In the seconds-to-minutes range, the dominant model of temporal processing has been the internal clock model^6,8^. This model suggests that a pulse count provides a linear metric of time and following temporal judgments rely on comparing the current pulse count to that of a reference time. Timing not only of longer intervals but also of intervals lasting from one second to tens of seconds appears consistent with mechanisms that generate a linear metric of time^6^. People with higher *internal clock speed* (ICS) perceive time passing faster than a stopwatch (e.g., for an objective 30 s period one might subjectively report 35 s elapsing). People with lower ICS perceive time passing slower than a stopwatch, i.e. subjectively reporting 25 s elapsing for an objective 30 s. There is a growing evidence that timing (or time perception) participates in value-based decision making, especially when temporal cues are available throughout the task, making time inherently more salient than many other stimulus dimensions in the intertemporal choice task^9^. Recent human and animal research has suggested that timing processes may play an important role in impulsive choice behavior^10–12^: priming with durations not only led to more precise time estimations, but also decreased subjects’ impulsive choices significantly.

Intuitively, there are a few ways that time perception might influence delay-discounting. First, the duration of time, like the value of money, is perceived logarithmically (or a similar decelerating function). The difference between 1 and 5 days seems to be more than the difference between 100 and 105 days. Some have argued that the form of deceleration of time perception may be related to the particular functional form of delay-discounting (i.e. hyperbolic versus exponential^13–15^). Second, individual differences in time perception can make the same duration feel short to one person and long to another. Imagine two people, Alice and Bob, who are waiting for their lunch orders. Allow that they are cognitively identical except that Bob has a faster internal clock than Alice, so after waiting 10 min, he feels like 20 min have passed. When asked if their lunches were worth the wait, Alice says ‘yes’ and Bob says ‘no’. Naively, we might think that Bob discounts time more steeply, but in fact, it was the difference in time perception, not discount rate, that caused the outcome. This is the intuition behind our study: subjects who have faster internal clocks should appear more impulsive, but only in the seconds task, where delays are experienced. While this imagined scenario might be intuitive, there are only a handful of investigations that have directly tested whether and how individual differences in time perception influence delay-discounting. Moreover, these studies have used hypothetical timing (i.e. “How long is 1 month?”) and hypothetical delay-discounting tasks which may limit their generalizability^16^. To date, we know only one study that has explored timing and intertemporal choice (both not hypothetical) in the seconds-to-minutes range^17^.

Here, we used both the delay-discounting tasks and also measured time perception and production. Our within-subject experiment directly addresses the question, posed by Wittmann and Paulus^18^, whether variation in timing in the seconds-to-minutes range influences not only discounting in seconds, but also discounting in days, weeks, and months. In our experiment, we fill the existing gap in the literature and compare timing on the range of seconds-to-minutes with delay discounting on the time-scale of seconds and of days, importantly, both not hypothetical: in the timing tasks subjects had to report perceived time and produce intervals and in the discounting task subjects’ payment depended on the choices they made.

Surprisingly, there is no consensus in the literature whether subjects with higher or lower ICS are more impulsive. As described above, intuitively, people with higher ICS should be more impulsive since, for them, time intervals are perceived subjectively as lasting too long^18–20^. However, several studies have failed to find this correlation^21,22^ and at least one study has found the opposite: that a higher internal clock speed is linked to lower impulsivity^17^. This counter-intuitive result may be explained via speculation that people with higher ICS might have faster processing speed^23^. Since choosing the later option is thought to involve extra cognitive processing and is robustly correlated with general intelligence^24^, faster processing speed should be linked to less impulsivity. In support of this, drift-diffusion modeling has linked higher ICS with ‘a more deliberate processing of the choice presented’^25^. Studies have also found that less accurate ICS is correlated with impulsivity^17^: the notion being that subjects who often experience large errors in their time estimation come to have a lot of uncertainty in predicting events. This uncertainty has been linked, theoretically and empirically to general avoidance of delayed options^26^.

One potential reason for the lack of consensus could be that there is substantial variation in intertemporal choice that is *not* influenced by ICS, making it statistically challenging to find a robust relationship. The strength of our design is that we tested subjects in two intertemporal choice tasks, only one of which should be influenced by ICS. Thus, we can use the long task (which should not be influenced by ICS) to effectively normalize the discount factor measured in the short task (which should be influenced by ICS). In this way, we have a novel and powerful way to test the relationship between ICS and intertemporal choice.

We had three main hypotheses about how the influence of ICS on the short intertemporal choice task could be measured. First, we hypothesized that subjects who appeared more impulsive in the seconds task than the days task (measured via the difference in discount factors in 1/s and in 1/day, respectively) would have faster internal clocks than those who appear less impulsive in seconds than days. Second, that the inter-subject variability in ICS would account for variability in discount factor in the short task that was unexplained by variability in discount factor in the long task. Third, if subjects’ delay-discounting choices in the short task were fit with a model that used subjective (based on performance in timing tasks) rather than objective time, then the correlation of the ‘subjective time’-short-discount-factor with the day-discount-factor would be higher than the correlation between ‘objective time’-short-discount-factor and the day-discount-factor.

We only found support for our second hypothesis: timing had additive value in explaining the time-horizons gap in discounting, but only when the time perception estimates were done at the same time as the decision-making choices and when the time perception task proxy for ICS was used. However, there was no evidence that subjects who were relatively impulsive in seconds vs. days had fast internal clocks (Hypothesis 1). Likewise, we did not significantly improve correlation between discounting on the time-scale of seconds and of days after accounting for subjective time (Hypothesis 3). Together, these results suggest that variation in ICS can contribute to small variations in estimated discount rates, but does not account for the unexplained variance between discounting in the seconds compared to days task and that the degree of contributed variation is small enough that it seems reasonable to ignore, at least for healthy participants.

## Results

**Figure 1:**
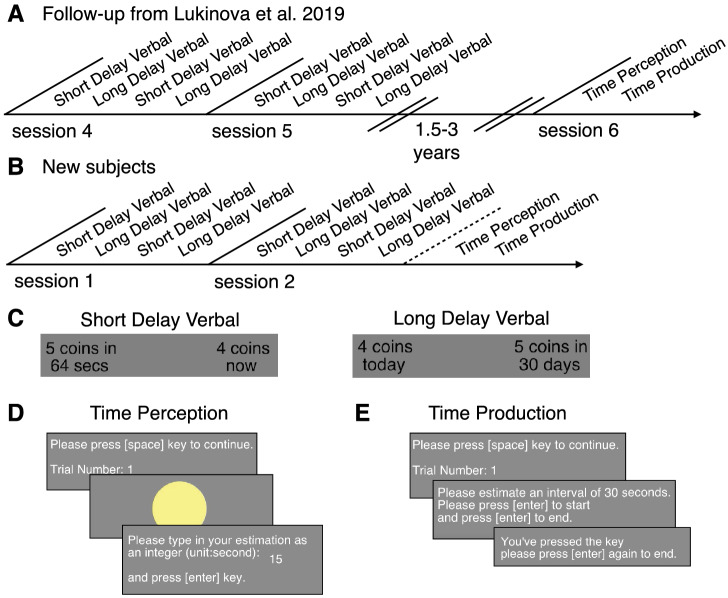
Behavioral tasks. (**A**) Timeline of experimental sessions for the follow-up group ($\hbox {n}=24$). During sessions 1–3 (not shown) subjects performed a non-verbal delay-discounting task (**B**) Timeline of experimental sessions for the new group ($\hbox {n}=26$). (**C**) Stimuli examples in the intertemporal choice task. (**D**, **E**) Screenshots of the timing experiment.

Two groups of subjects in this study participated in intertemporal choice tasks to estimate discount factors and timing experiments to estimate internal clock speeds. The follow-up group had a greater than 1 year gap between the intertemporal choice tasks and the timing experiments (Fig. 1A). The new subjects performed the timing experiments on the same day as their second session of intertemporal choice tasks (Fig. 1B).

### Delay discounting

In each trial of the delay-discounting task, subjects made a decision between a sooner and a later option (Fig. 1C; as in Lukinova et al.^5^). Two delay-discounting tasks were considered: the verbal short delay task (SV, or the seconds task) where the delays to reward were in seconds and the verbal long delay task (LV, or the days task) where the delays were in days. Importantly, in the SV task, in each trial that the subject chose the later option, they experienced the delay. In contrast, in the LV task, after the choice the next trial proceeded immediately. At the end of each LV session, one trial was chosen at random. If the subject chose the later option on that trial then an electronic payment was delivered after the chosen delay.

Subjects’ discount factors were estimated by fitting their choices with a Bayesian hierarchical model (BHM) of hyperbolic discounting with decision noise (as in Lukinova et al.^5^). The model (described as the ‘objective time model’ in “Methods/Analysis”) had four population level parameters: log discount factor, $\log (k)$, and the log of the decision noise, $\log (\tau )$ for both intertemporal choice tasks; and three parameters per subject: $\log (k_{SV})$, $\log (k_{LV})$ and $\log (\tau )$. We used this model to fit 10,269 choices across 26 subjects in the new group. We re-used the fits from Lukinova et al.^5^ for the follow-up group.

**Figure 2:**
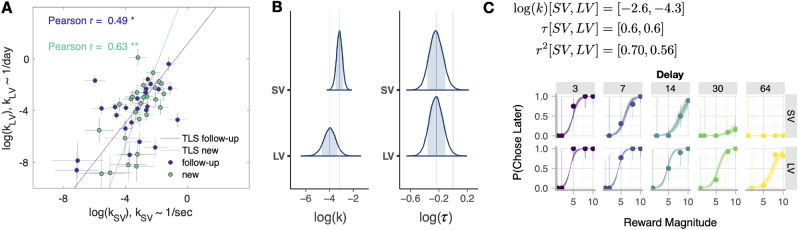
Comparison of discount factors across groups and tasks. (**A**) Each circle represents one subject ($N=50$). The log discount factors in short delay verbal task (SV, x-axis) plotted against the log discount factors in long delay verbal task (LV, y-axis). Discount factors were estimated in the units of the task. The color of the circles identifies the group, either follow-up (purple) or new (cyan). The error bars indicate the standard deviations of the log discount factor. The lines represent the total least squares (TLS) regression lines for two groups^27^. (**B**) Distribution of posterior parameter estimates of $\log (k)$ and $\log (\tau )$ from the model fit of the new group of subjects. (**C**) Intertemporal choices and softmax-hyperbolic fit of one example subject from the new group. In each panel, the marker and error bar indicate the mean and binomial confidence intervals of the subject’s choices for that offer. The smooth ribbon indicates the BHM model estimates (at 50, 80, 99% credible intervals). Each column shows the choices for a specific delay (in seconds for SV, top row; in days for LV, bottom row). At the top of the subject plot we indicated the mean estimates of $\log (k)$, $\tau$ and the Bayesian $r^2$ for each task for that subject.

Consistent with our previous result, we found strong intersubject correlations between the seconds and days task. In other words, the most patient subject in the seconds task was likely to also be patient in the days task. This was true for the new group (Fig. 2A; Pearson $r = .63$, $p<.001$) and for the follow-up group (Pearson $r = .49$, $p = .014$). We also found that the population level parameters of the fit to the new group were similar to our previous results (compare Fig. 2B with figure 3C in Lukinova et al.^5^). All subjects’ choices were well-fit by the objective time model (Fig. 2C for an example subject and SI Fig. S1 for all subjects).

### Time estimation and production

**Figure 3:**
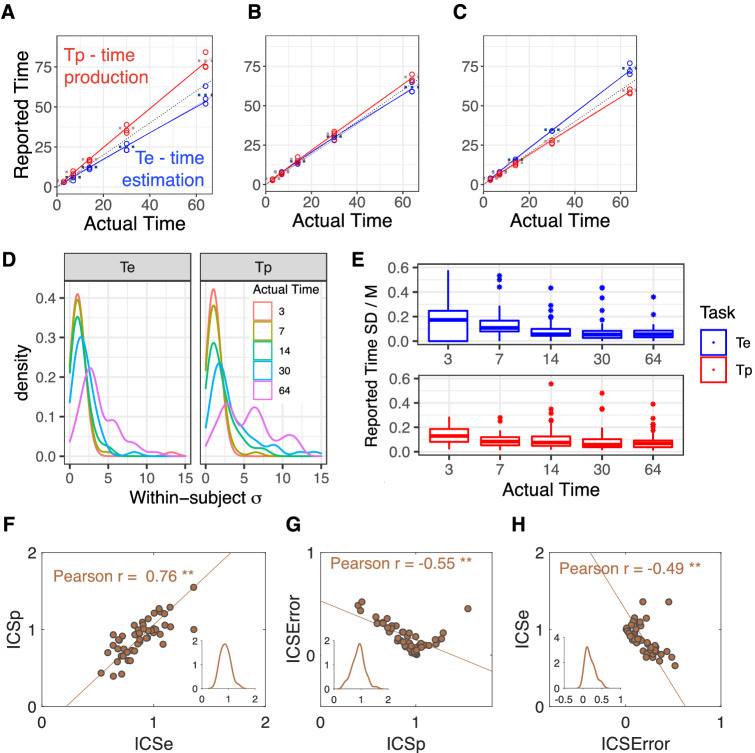
(**A**–**C**) Reported versus actual time for three example subjects. Circles correspond to individual trials and the horizontal dotted line shows the mean per interval (Production, red; Estimation, blue). $y=x$ is drawn in dotted gray. The solid and dashed lines of each color are the power and the linear fits, respectively. If the estimation time was slower (blue below $y=x$) and the produced time was faster (red above $y=x$) than a stopwatch that was consistent evidence for a lower ICS. A subject with (**A**) low ICS, (**B**) accurate ICS & (**C**) high ICS. (**D**) Subjects showed scalar timing. Distribution of within-subject standard deviations grouped by the type of timing task and actual time interval ($N=50$ subjects in each density plot with outlier trials removed, “Methods”). See also Fig. S5 and Table S1 in SI. (**E**) Boxplots of SD/M for each duration. The SD/M was stable across durations, consistent with scalar timing (Production, red, two outlier points were truncated; Estimation, blue, eight outlier points were truncated). (**F**–**H**) Scatter plots ($N=49$) between *ICSe*, *ICSp* and ICS error with TLS regression lines. Significant correlation between *ICSe* & *ICSp* measures indicates that the tasks reliably captured between subject variability in ICS. Inset: kernel density estimation of respective timing variable (x-axis).

Subjects internal clock speeds (ICS) were estimated using two tasks: a time perception (estimation) task where subjects reported the duration that a visual stimulus appeared on the screen (Fig. 1D) and a time production task where subjects were presented with an interval and they had to start and stop the indicated interval with keypresses on the keyboard (Fig. 1E). Subjects’ performance was well described by both linear and power fits for both estimation and production (Fig. 3A–C shows three example subjects; see Fig. S4 for all subjects). The variation in timing increased with longer intervals for both estimation (Te) and production (Tp) tasks (Fig. 3D), a key signature of scalar timing, which “requires timing sensitivity to remain constant as durations timed vary”^28^. This can also be seen by plotting the SD/M for each interval (Fig. 3E). As expected, the coefficient of variation (SD/M) remained roughly constant, consistent with Weber’s law^29^. This is in stark contrast to data from subjects’ using a counting strategy, where the standard deviation is constant as a function of interval (see Fig. 5, intervals $> 1$ s in Grondin et al.^30^).

Two proxies (*ICSe* and *ICSp*) for ICS were calculated using Eqs. (1) and (2), respectively (“Methods”). The majority of our subjects had a lower ICS, meaning that they would perceive time passing slower than a stopwatch (e.g., for an objective 30 s period an individual might subjectively report 25 s elapsing). The strong correlation between proxies for internal clock speed supports our choice for ICS as a reliable measure of timing: correlation between *ICSe* and *ICSp*, Pearson $r = .76$ , $p < .001$ for all subjects in Fig. 3F. The ICS error was calculated by capturing distortions from accurate timing (Eq. 3) and was negatively correlated with both *ICSe* and *ICSp* (Fig. 3G, H). The follow-up and the new group were not significantly different according to permutation tests (SI, Individual timing) for each of the three timing variables.

We fit each subjects’ estimation and production raw data (separately) with power and linear functions (Eqs. 6, 7, respectively) of the actual time. All subjects’ subjective timing was fit well (using a BHM, SI, Subjective time estimation) with both linear and power functions. The exponent of the power function (Eq. 6), $\beta$, was close to 1 for many subjects so the power and the linear fits overlap (Fig. 3A–C and SI Fig. S4). According to 10-fold cross validation criteria (‘kfold’ model comparison in SI Table S2) time estimation was better fit with a power function and time production with a linear function. Therefore, for the subjective time model further on we used both linear and power fits.

### Timing explains variation in discounting only when measured proximally

**Figure 4:**
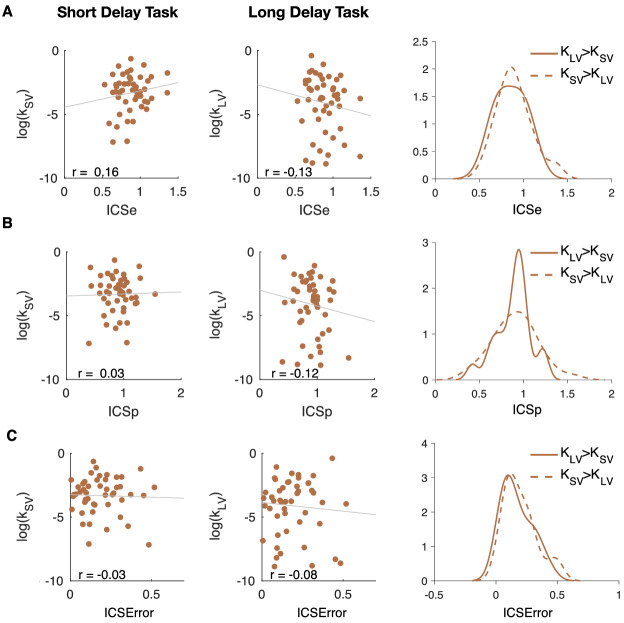
(**A**–**C**) First two columns represent correlations between discount factors (in short delay and long delay tasks, y-axis) with *ICSe*, *ICSp*, and *ICSError* (x-axis). Each circle is one subject ($N=49$). Pearson’s *r* is reported on the figure (all $p > .05$). Best linear fit line ($y \sim x$) is displayed. There was no evidence of correlation between any ICS measure and discount factor. The rightmost column represents kernel density estimations of *ICSe* and *ICSp* as proxies for ICS and *ICSError* split by positive or negative difference between discount factor in short and long delay tasks. There was no significant difference in internal clocks between subjects who appear more impulsive in the seconds task than the days task ($K_{SV}>K_{LV}$) and those, who appear more impulsive in the days task than the seconds task ($K_{LV}>K_{SV}$). These results did not support our first hypothesis that subjects who appear more impulsive in the seconds task than the days task will have fast internal clocks and vice versa.

Having estimated subjects’ time preferences and timing properties we tested for correlations between them. We found no significant relationships between *ICSe*, *ICSp*, *ICSError* and discount factor ($N=49$, both jointly in Fig. 4 and within each group in SI Fig. S6A–C). This lack of correlation stands in contrast to previous literature that found a positive correlation between *ICSError* and impulsivity^17^. Furthermore, when we separated subjects who appear more impulsive in the seconds task than the days task and vice versa, we did not find that the groups were significantly different from each other. We observed no differences in *ICSe*, *ICSp*, *ICSError* distributions when split by the sign of the difference between discount factor in delay-discounting tasks (Fig. 4A–C; *ICSe* permutation test between $K_{LV}>K_{SV}$ and $K_{SV}>K_{LV}$ subgroups: $M_{K_{LV}>K_{SV}} = 0.85$ and $M_{K_{SV}>K_{LV}} = 0.90$ with $p = .444$; *ICSp*: $M_{K_{LV}>K_{SV}} = 0.89$ and $M_{K_{SV}>K_{LV}} = 0.91$ with $p = .787$; *ICSError*: $M_{K_{LV}>K_{SV}} = 0.1698$ and $M_{K_{SV}>K_{LV}} = 0.1961$ with $p = .492$, where *M* indicates the mean; see SI for permutation tests for *ICSe* separately for the two groups). Using the sign of the residuals of the total least squares fit (in Fig. 2A) to split our sample into subgroups did not change these results. Thus, we failed to find support for our first hypothesis: that subjects who were more impulsive in the short compared to long delay-discounting task would have higher ICS than subjects who were more impulsive in the long compared to the short task.

**Figure 5:**
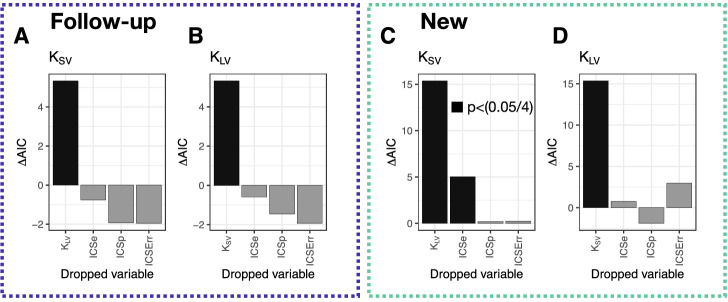
Drop-one regression analysis. We generated linear regression models of $\log (k)$ for each task (short delay and long delay) against the discount factor of the other task, as well as timing variables. In order to test which factors were important, we dropped each factor and tested whether the decrease in likelihood was significant by a $\chi ^2$ test. (Analyses were done in R using the ‘drop1’ function). We plot the change in AIC, with significant drops in black ($p<.0125$, Bonferroni Corrected $p<.05/4$). (**A**, **B**)—follow-up group; (**C**, **D**)—new group. Only for the new group and with *ICSe* we found some evidence that subjects’ timing was related to their discount factors. Dropping this proxy for ICS resulted in a significant decrease in the likelihood for explaining short delay.

We demonstrated that discount factors were significantly correlated between the short and long task (Fig. 2A). Here, we tested our second hypothesis: whether timing might account for variance in discount factor in the short task beyond what was explained by the discount factor in the long task (but not vice-versa). To this end, we ran linear regressions according to Eqs. (4) and (5). We tested the contribution of each factor by dropping it from the model to create a reduced nested model and performing a likelihood ratio test against the full model ($N=49$, Fig. 5). We found that subjects’ timing in the new group, but not in the follow-up group, was related to their discount factors. Dropping *ICSe* (a proxy for ICS) resulted in a significant decrease in the likelihood for explaining short delay ($N=26$, Fig. 5C), but not long delay task in the new group. Thus, we found some support for our hypothesis that ICS accounts for some of the variance in delay discounting for short experienced delays (but only for time estimation and the new group). With the addition of *ICSe* we explained 48% (an increase in 8% compared to 40% for reduced model) of the variance in $\log (k_{SV})$ (Table 1). Detailed results of the remaining regression models were presented in SI Tables S2–S4. In the joint group analysis, dropping *ICSe* did not significantly decrease the likelihood for predicting short delay task choices, suggesting that the effect was really only for the ‘new’ group. Still, adding *ICSe* resulted in an increase of 5% of variance explained (SI Tables S6 and S7).

**Table 1:** New Group $\log (k_{SV})$ Regression Results. This regression analysis was done according to Eq. (4).

	*Dependent variable:*$\log (k_{SV})$		
	(1)	(2)	(3)
$$\log (k_{LV})$$	$$0.313^{**}$$	$$0.286^{**}$$	$$0.300^{**}$$
	(0.070)	(0.072)	(0.069)
ICSe	$3.364^{*}$		1.671
	(1.318)		(0.875)
ICSp	− 1.559		
	(1.149)		
ICSError	2.019		
	(1.475)		
Constant	$$-3.872^{**}$$	$$-2.001^{**}$$	$$-3.457^{**}$$
	(0.888)	(0.342)	(0.829)
Observations	26	26	26
$\hbox {r}^{2}$	.567	.397	.480
Adjusted $\hbox {r}^{2}$	.484	.372	.435
Residual Std. Error	0.806 (df = 21)	0.889 (df = 24)	0.844 (df = 23)
F Statistic	$$6.865^{**}$$ (df = 4; 21)	15.822$$^{**}$$ (df = 1; 24)	10.607$$^{**}$$ (df = 2; 23)

By looking at the $r^2$, we found some support for our hypothesis that ICS accounts for some of the variance in delay discounting for short experienced delays. With the addition of time estimation proxy for ICS we explained 48% (an increase in 8% compared to 39.7% for reduced model) of the variance in $\log (k_{SV})$.

$$^{*}\hbox {p}<.05$$; $$^{**}\hbox {p}<.01$$.

### Accounting for subjective timing does not improve fits nor does it change the correlation between discount factors significantly

If individual differences in ICS influenced choices in the short delay-discounting task, then using subjective time (*ST*), rather than objective time should improve our ability to predict subjects’ choices. To this end, we compared the model with objective delays (‘obj’) to four models with subjective delays:

- ‘subjTep’—where delays in seconds were substituted by the power fits based on time estimation,
- ‘subjTel’—where delays in seconds were substituted by the linear fits based on time estimation,
- ‘subjTpp’—where delays in seconds were substituted by the power fits based on time production, and
- ‘subjTpl’—where delays in seconds were substituted by the linear fits based on time production.

After accounting for subjective timing in the short delay task we did find a higher (but not significantly higher according to ‘cocor’ tests, “Methods”) correlation between discount factors in the short and long delay tasks (Table 2). This was only true in the models that replaced objective time with subjective time based on the time estimation task (both within each group and for groups combined in SI Table S8). Therefore, we only found nominal evidence to support our third hypothesis: adjusting for individual heterogeneity in timing did not improve the correlation between discounting factors across seconds and days.

**Table 2:** Pearson correlation between $\log (k_{SV})$ and $\log (k_{LV})$.

	Pearson *r* value	*p* value	Significantly better?
**New**			
obj	.63	< .001	
subjTel	.64	< .001	No
subjTep	.64	< .001	No
subjTpl	.51	.008	No
subjTpp	.51	.007	No
**Follow-up**			
obj	.43	.034	
subjTel	.48	.016	No
subjTep	.48	.018	No
subjTpl	.39	.063	No
subjTpp	.41	.046	No

‘Significantly Better?’ answers whether there has been a significant increase in correlations from the objective time model to the respective subjective time model tested using R package cocor (“Methods”).

After accounting for subjective timing in the short delay task we hypothesized to find a higher correlation between discount factors in the short and long delay tasks. However, although we found stronger correlations none of them resulted in significant increase, thus, not supporting our third hypothesis.

## Discussion

Scholars have argued that it is crucial to combine timing and intertemporal choice research^11,18,31,32^. Here we measured one aspect of timing, internal clock speed, estimated using time estimation and production tasks to test whether variation in ICS explains variation in subjects intertemporal choices between tasks where the delays are experienced versus not experienced. We hypothesized that subjects with fast ICS would experience delays as longer and thus be less willing to wait for experienced delays. In this paper, we have shown that accounting for subjects’ timing helps to predict discounting, but only using variation in time perception to explain variance in short delay tasks and when timing and discounting are proximally assessed. In the new group of subjects, accounting for timing explained an additional 8% of variance in discount factors in the seconds task: an effect which supported our second hypothesis. However, we did not find support for our other hypotheses that larger errors in timing are associated with more impulsive subjects in the short delay task, nor did we find a significant increase in correlation between short and long delay discount factors after accounting for subjective timing.

Our findings highlighted the importance of temporal proximity of the timing and the intertemporal tasks. One possible explanation for the difference in results between the new and the follow-up groups (Fig. 5A vs. C) was that similar to how a timing task can influence the performance of the following discounting tasks^10,33,34^, the discounting task in the new group influenced performance on the subsequent timing task. However, since the distributions of internal clock speeds in the new and the follow-up groups were highly overlapping, this explanation was unsupported by the data. Another possible explanation for this difference was that both internal clock speed and discounting were somewhat unstable over time. For example, both timing and discounting can be perturbed by emotional state^35,36^ or caloric intake^37,38^. These, and other, factors could contribute to moderate test-retest reliability for discounting^39^ or timing^40^ which would, in the follow-up group, wash out the small effects observed here in the new group.

There are many ways to assess ICS. In this paper we used just the time perception and the time production tasks on the time range of seconds to minutes and three different measures of internal clock speed, without a strong a priori belief about which would influence choices in the delay-discounting task: time perception internal clock speed (*ICSe*), time production internal clock speed (*ICSp*), or internal clock speed errors (*ICSError*). We found only time perception (*ICSe*) to be associated with discounting in the seconds task. Sensory timing (duration discrimination, perception) and motor timing (production, reproduction of the time interval) putatively have distinct underlying neural mechanisms^41^. We note that, in both the time perception and the intertemporal choice tasks, time was controlled by the experimenter which makes these two experiments closer to each other, in comparison to the production task where timing was controlled by the subject. Moreover, time perception seems to be intertwined in the short delay task: you may experience the delay in seconds once, but then decide to wait or not on another similar trial (update your belief) based on your subjective experience of the elapsed time. Unlike in the time production experiment, where time was controlled (started and ended) by the subject, in the short delay task subjects were not able to give up waiting. As van den Broek et al.^19^ puts it, production tasks involve not only temporal judgement but also an ability to withhold a response: taking an action to indicate the end of the interval. Therefore, time production performance may not be indicative of “deficient temporal discrimination per se, since their performance might be attributable to difficulty in inhibiting responding”^3^.

Overall, we did not find a strong relationship between timing and intertemporal choice. It is worth noting that the previous studies investigating the relationship between these two phenomena sometimes found positive^20^, sometimes found negative^17,25^ and sometimes found no correlations^21,22^ between internal clock speed and discount factors. In contrast to previous published work, we did not find internal clock speed errors (*ICSError*) to be correlated with the discount factors in the sample of college students. We speculate that a clinical sample with high levels of impulsivity might be a better one to relate to a less accurate ICS (higher *ICSError*) as previous works suggest: to an impulsive person the passage of time may appear to be more intolerable and more error prone^41^. For example, research on children (in the context of attention-deficit/hyperactivity disorder or preterm birth) suggests that there is a positive relationship between waiting time in the delay of gratification task and children’s performance in the timing task^42–45^. Also, animal research finds that the delay-exposure training in rats significantly decreases not only the number of impulsive choices, but also alcohol consumption level^12^. Taken together, these results reinstate the importance of core timing processes in impulsive behaviors^10^.

Considering our preregistered findings along with the inconsistency in the direction of the relationship in previously published studies, several conclusions are possible. First, there may be no reliable relationship between individual differences in timing and intertemporal choice, despite the intuitive appeal of such a relationship. Alternatively, there is a subtle relationship, but it depends on poorly understood contextual factors that have obstructed replication across studies. To definitively resolve this question, a larger preregistered multi-site study (similar to^46^) should be done including both neurotypical and clinical populations, where more extreme examples of individual differences in timing and intertemporal choice might reveal a connection.

## Methods

This study was preregistered at OSF (https://osf.io/vaqf8). Unless otherwise specified, all experimental methods and analyses presented here were as described in the preregistration.

### Participants

This study included two groups of subjects: a ‘follow-up’ group (19 women and 5 men that participated previously in the main or control experiment 1 of Lukinova et al.^5^ and a new (named ‘naive’ in the preregistration) group of subjects (Fig. 1A, B). We recruited new subjects for two reasons. First, we anticipated the challenge of bringing back the desired number of subjects. Second, we anticipated that an individual’s variability in timing or delay-discounting over the course of a year might overwhelm a small relationship between timing and discounting (since our initial delay-discounting study took place more than a year before the data collection for this study).

For the new group, 30 participants were recruited from the NYU Shanghai undergraduate student population. Four subjects were excluded from all analyses, because their choices were insensitive to delay, leading to a total sample of 26 students (16 women, 10 men). The study was approved by the IRB of NYU Shanghai. All human subjects research at NYU Shanghai is conducted in accordance with the US policy and regulations found in 45CFR46, as well as in accordance with Chinese policy and regulations found in Measures for the Examination of Ethics for Biomedical Research Involving Humans. In the event of conflict between applicable standards of protection, NYU Shanghai follows the standard that provides greater protection to human subjects. Each participant gave written informed consent for participation in the study at the start of the first session.

The subjects were between 18–21 years old and 13 subjects were Chinese nationals. Subjects received a 40 CNY ($$\sim$$ $5.5 USD) per hour participation fee as well as up to an additional 50 CNY ($$\sim$$ $8 USD) per session based on their individual choices in the delay-discounting tasks. There was no monetary incentive to be accurate in the timing tasks. For the follow-up group, there were two sessions of intertemporal choice (2 weeks apart from each other) and after 1.5-3 years, a third session of timing tasks (Fig. 1A). For the new group, there were two sessions (2 weeks apart from each other): both sessions included intertemporal choice tasks and the second session also included timing tasks (Fig. 1B). All sessions (for both groups) took place in the NYU Shanghai Behavioral and Experimental Economics Laboratory in Shanghai. All decisions in the intertemporal choice task involved a choice between a later (delays in seconds and days) option and an immediate (now) option (Fig. 1C).

Corvi et al.^17^ reported moderate correlations between discount factors and timing variables such as internal clock speed error ($r = .43$, $p<.01$), and $r = -.31$ for internal clock speed. Thus, we expected an *r* value from .3 to .5 for the correlation between timing variables and discount factor in seconds. A power analysis indicated that for expected correlation $r = .5$ and 80% power (the ability of a test to detect an effect, if the effect actually exists) the required sample size was $N = 29$, for a medium size correlation of $r = .3$ the required sample size was $N = 84$^47^. We preregistered collection and analysis of data from 30 participants in each experimental group. However, we were able to bring back only 24 participants for follow-up group (19 women, 5 men; between 20 and 24 years old; 14 subjects were Chinese Nationals). Therefore, a total of 50 (out of 54) subjects with 30 (5 time interval $\times$ 3 repetitions $\times$ 2 tasks) timing observations per each subject was considered for analysis. This was a within-subject study, so all conditions applied equally to all subjects.

### Materials and procedure

In our timing experiment, we used two tasks to measure each subject’s timing properties (Fig. 1D–E). They were always in order: first, a time perception task, then, a time production task. We used this order since the time intervals for estimation were the same as those for production and we did not want to bias our subjects with any numbers prior to assessing their time perception. All subjects first participated in a demonstration with instructions and two trials of each task (interval = 10 s) to facilitate the real experiment.

For the time perception task the instruction was: “Welcome to the experiment. You will estimate how long the circle has shown up and type in your estimation as an integer (unit = seconds). Please press [space] key to start the experiment when you are ready.” The time intervals (*t*) that were used in the task were 3, 7, 14, 30, and 64 s, each repeated three times, in random order.

For the time production task the instruction was: “Welcome to the experiment. You will press the [enter] key to start and press the [enter] key to end the estimation of the given time intervals. Please press [space] key to start the experiment when you are ready.” The time intervals (*t*) that were used in the task were 3, 7, 14, 30, and 64 s, each repeated three times, in random order.

We repeated each of the timing intervals presentation three times in our timing tasks following the design from Corvi et al.^17^. Although some experimenters point to a repetition effect affecting the time estimates, according to Matthews^48^, effects disappear when there is a modest lag between presentations (in our case, five different stimuli in random order). Also, Miomi et al.^49^ revealed that time production techniques are not equivalent, with the method involving key presses to start and stop the production (which we used) showing the highest accuracy. Contrary to Rattat and Droit-Volet^29^, in both timing tasks we did not provide instructions ‘not to count’. In earlier pilot studies, subjects reported that this instruction was hard to follow (e.g. forced some subjects to shift attention away from the timing task to stopping themselves from counting) and resulted in unreliable data that did not adhere to scalar timing properties. That said, analyses of the data are consistent with scalar timing, suggesting that the subjects did not count. Both tasks took approximately 12–15 min to finish. The duration of each task (not including the real timing distortions and intertrial intervals that depend on each subject’s speed) was $(3+7+14+30+64) \times 3/60 = 5.9$ min. Subjects received a fixed payment of 40 CNY for both timing tasks.

The intertemporal task for new participants mimicked the last two sessions in Lukinova et al.^5^ control experiment 1 ‘no circles’ (Fig. 1C). Two sessions (two weeks apart) included an alternating set of verbal tasks (verbal short delay, SV, and verbal long delay, LV; SV-LV-SV-LV in Fig. 1B or LV-SV-LV-SV, for a random half of subjects). For the short delay tasks, when subjects chose the later option, a clock appeared on the screen, and only when the clock image disappeared, could they obtain their reward, visualized as a stack of coins. The visual presentation of coins was accompanied by a ‘dropping coins’ sound. The payment was done differently for SV and LV: in the former, subjects accumulated coins and the total earned was paid via electronic payment at the end of each experimental session, in the latter, a single trial was selected at random at the conclusion of the session for payment. In our sessions, the exchange rate in SV was 0.05 CNY per coin (since all coins were accumulated and subjects were paid the total profit), whereas in LV, the exchange rate was 4 CNY per coin. These exchange rates were set to, on average, equalize the possible total profit between short and long delays tasks. In each trial, irrespective of the task, subjects made a decision between the sooner and the later options. The sooner option was fixed at 4 coins now (or today). There were 25 different later options presented in each task, i.e. all possible combinations of 5 delays and 5 reward magnitudes: 3, 7 (6.5 for the follow-up group), 14, 30, 64 s (or days) and 1, 2, 5, 8, 10 coins, respectively. Across sessions, 200 trials were conducted in verbal short delay (SV, delays in seconds) and verbal long delay (LV, delays in days).

### Analysis

#### Preregistered analysis

Timing experiments resulted in several observations per subject in the time perception and time production tasks. Some researchers used only a production task and calculated the internal clock speed (ICS) as the ratio of produced versus actual duration^50^. Since we used two tasks we converted observations from both tasks into our key variables following the procedure from Corvi et al.^17^.

First, per each time interval (*t*) and each subject, the average time estimation (*Te*(*t*)) and average time production (*Tp*(*t*)) were calculated by averaging reported time in three trials for the same time interval and the same subject. Then, the ratios *TeRatio*(*t*) and *TpRatio*(*t*) were calculated by dividing *Te*(*t*) and *Tp*(*t*) by the actual time interval duration (*t*) per subject, respectively as suggested by Glickson and Hadad^50^. Next, we adjusted the procedure, since Corvi et al.^17^ used only one interval per task, and averaged the ratios across time intervals in each task per subject (*MTeRatio* and *MTpRatio*).

In general, people with higher than average ICS estimate an objective duration to be longer than average and tend to produce shorter durations than average^8^. We decided to separately consider data from the production task and the perception task as proxies for ICS. According to the definition, the calculation of the proxy for ICS per subject from time perception (estimation) task was straight-forward:

1 $$\begin{aligned} ICSe = MTeRatio \end{aligned}$$

Thus, ICS values higher and lower than 1 indicate internal clock speeds faster and slower than objective time, respectively. For the production task we had to symmetrically reflect the value of the averaged timing ratio around 1 in order to adhere to the same meaning of the ICS. So, for those subjects whose $MTpRatio>1$ the proxy was calculated as $ICSp = (1 - abs(MTpRatio-1))$ and, otherwise, $MTpRatio<1$ the proxy was calculated as $ICSp = (1 + abs(MTpRatio-1))$ (the latter definition was preregistered). Combined, this gives us the equation below for the time production proxy for ICS:

2 $$\begin{aligned} ICSp = 2 - MTpRatio \end{aligned}$$

The ICS error per subject was calculated as:

3 $$\begin{aligned} ICSError = (abs(MTeRatio - 1) + abs(MTpRatio - 1))/2 \end{aligned}$$

thus, higher values indicate greater error.

We called the variables *ICSe*, *ICSp*, and *ICSError*, as defined above, the ‘timing variables’ per subject. For plotting the distributions of the timing variables (Fig. 3E–G) we calculated probability density estimates (for smoothing) using the ksdensity function in Matlab. By default the estimate is based on a normal kernel function, and is evaluated at equally-spaced 100 points, $x_{i}$, that cover the range of the data in *x*.

In the preregistration, we did not expect subjects’ time production to be distorted more than two times. However, one of our subjects produced an interval of more than 160 s when asked to produce 64 seconds. In this case, Eq. (2) cannot convert the *MTpRatio* to a proxy for ICS correctly. This subject did adhere to the scalar properties and was not removed from the overall analyses. However, whenever proxies for ICS and *ICSError* were used, this subject’s data were removed. Also, in the scalar timing analysis we removed 19 points representing subjects trials (outliers) in which they exceeded $3 * mean$ for that Actual Time interval.

In order to test our hypotheses, we compared timing variables to the discount factors in short and long tasks. The discount factors for the new subjects were estimated using a softmax-hyperbolic fit in a similar way as in Lukinova et al.^5^, i.e. a four population level and three subject level parameters model (mixed-effects model) was used with the help of ‘brms’ package in R^51^ that allowed to do a Bayesian hierarchical model (BHM) of nonlinear multilevel models in Stan with the standard R formula syntax^52,53^. Objective time model:

- choice$\sim$inv_logit((later_reward/(1 + exp(logk)*delay)-sooner_reward)/exp(logtau)),
- logtau$\sim$task + (1 | subjid),
- logk$\sim$task + (task | subjid)

where later_reward was the later reward, sooner_reward was the sooner reward; logk was the natural logarithm of the discounting parameter *k* and logtau ($\log (\tau )$) was the log of the decision noise. Fitting $e^{\log (k)}$ allowed $\log (k)$ to vary from $-\infty$ to $+\infty$ while $k=e^{\log (k)}$ was restricted to [0,$+\infty$]. For the subjective time models we substituted delays in seconds by power or linear fits based on two timing tasks (four subjective time models total). All models ($M_{4p,3s}$) had 4 population level parameters ($\log (k)$ and $\log (\tau )$ for each of the two intertemporal choice tasks) and 3 parameters per subject: $\log (k_{SV})$, $\log (k_{LV})$ and $\log (\tau )$. We used a normal prior for $\log (k)$ parameter with mean − 5 and standard deviation of 3 and a normal prior for $\log (\tau )$ parameter with mean 0 and standard deviation of 0.3 based on our expectations from previous studies in delay discounting. By default brms utilizes the No-U-Turn Sampler (NUTS^54^) implemented in Stan. All models were fitted using 10 chains, each with 6000 iterations of which the first 2000 were warmup to calibrate the sampler, leading to a total of 40,000 posterior samples. R package shinystan^55^ was used to diagnose and develop the models.

Following our general hypotheses, we examined whether there were:

- a significant difference in terms of subjects’ ICS for those who were more impulsive in the short compared to the long delay-discounting task (and vice versa) and a positive correlation between *ICSError* and discount factor (bigger error $\sim$ more impulsive) for short delays;
- individual differences in short verbal discount factor, but not long verbal discount factor, that were accounted for by differences in timing variables;
- a higher correlation between discount factors in short and long delays after accounting for subjective timing in the short delay task.

We used the following linear models to test the contribution of timing variables to each discount factor:

4 $$\begin{aligned} \log (K_{SV}) \sim ICSe + ICSp + ICSError + \log (K_{LV}) \end{aligned}$$

5 $$\begin{aligned} \log (K_{LV}) \sim ICSe + ICSp + ICSError + \log (K_{SV}) \end{aligned}$$

This regression analysis addressed the second hypothesis. R package lme4 was used for linear models^56^.

To convert objective time into subjective time we considered both power and linear forms of subjective timing (see fitting details in the SI, Subjective time estimation). The functional form for the power law was

6 $$\begin{aligned} ST(t)=\alpha \cdot t^{\beta } \end{aligned}$$

where *ST* was the subjective time, *t* was the target (actual) duration, $\alpha$ was a linear scaling in producing (or estimating) durations, and $\beta$ captured the degree of nonlinearity^14,50^. We also planned the estimation of a special case of the power function, where $\beta = 1$.

7 $$\begin{aligned} ST(t) = \alpha \cdot t \end{aligned}$$

Then $\alpha$, the slope of a linear function, reflected the change in the produced (or in the estimated) duration for a unit change in the target duration (considered as another index of ICS^18,25^). For a time production task, the higher the slope the more time was produced for a unit change in the target duration reflecting a lower ICS.

Other planned analyses, reported in the SI, included performing nonparametric tests to compare timing variables between genders and correlations of timing variables with the Barratt Impulsiveness Scale (BIS).

The permutation tests of differences between the means of two groups were done by shuffling the group label and computing the mean between the shuffled groups 10,000 times. This generates a null distribution which was used to estimate the probability of observing the true difference between groups (bootmean in https://github.com/erlichlab/elutils).

The significant difference in correlations (e.g., between the objective model and four subjective time models) was tested using R package cocor^57^ assuming nonoverlapping dependent correlations.

#### Additional analyses

As mentioned in *Participants*, the follow-up group was only a subset of the subjects from Lukinova et al.^5^. As we were using a hierarchical model to estimate the parameters of each subject, the inclusion or exclusion of other subjects can subtly influence the estimates of any individual subject. For the analyses reported in Table 2 ‘follow-up’, we fit those models using only the follow-up group. The correlation coefficient between short and long discount factor for these subjects based on the original model in Lukinova et al.^5^ (Pearson $r = .49$) was not significantly different (using the ‘R cocor’ tests) from the one reported here (Pearson $r = .43$). We also fit the objective time models for the follow-up and the new groups combined. The discount factors aligned well across the fits (see SI Fig. S3).

In order to check whether the relationship between timing and discounting was present regardless of the group and to increase the statistical power, we re-ran all our analyses with the follow-up and the new groups combined (not preregistered). Some of the joint results were listed in the main text, others were reported in SI (Post hoc analysis). Importantly, all results before and after combining the samples were consistent.

### Software

The code for the timing experiment was written in Python using the ‘PsychoPy’ toolbox (version 1.83.04^58^). The code for the intertemporal choice task was re-used from Lukinova et al.^5^ available at https://www.github.com/erlichlab/delay3ways. All analyses and statistics were performed either in Matlab (version 9.3, or higher, The Mathworks, MA), or in R (version 3.4.1, or higher, R Foundation for Statistical Computing, Vienna, Austria^59^). R package brms (2.0.1) was used as a wrapper for rstan to perform Bayesian nonlinear multilevel modeling. R package stargazer^60^ was used to transform R regression results to LaTex tables.

## Supplementary Information

Supplementary Information.

## Data Availability

The code, de-identified raw data and saved model fits necessary for regenerating main results and figures are available as a Zenodo release (https://doi.org/10.5281/zenodo.5198167) of a GitHub repository (https://github.com/erlichlab/delayTP/).
