# Supplementary material for: Quantifying the contribution of individual variation in timing to delay-discounting
Source: Sci Rep. 2021 Sep 15;11:18354. doi: 10.1038/s41598-021-97496-w (PMC8443764; doi:10.1038/s41598-021-97496-w)
Supplement: Supplementary file 1 — Supplementary Information. [file 41598_2021_97496_MOESM1_ESM.pdf]

Quantifying the contribution of individual variation in timing to delay-discounting

Evgeniya Lukinova[1,2] **and Jeffrey C. Erlich**[1,2,3,*]

[1]NYU-ECNU Institute of Brain and Cognitive Science at NYU Shanghai, Shanghai, 200062, China
[2]NYU Shanghai, Shanghai, 200122, China
[3]Shanghai Key Laboratory of Brain Functional Genomics (Ministry of Education), East China Normal University, Shanghai, 200062, China
[*]jerlich@nyu.edu

Supplemental Information

Individual subject fits and re-groupings in intertemporal choice

Consistent with our previous results, the new subjects were well-fit by the BHM model in the delay-discounting task (Figure S1, multi-page PDF is available at github). The follow-up group was a subset of the subjects that previously participated in the intertemporal choice experiments in Lukinova et al.[1]. Throughout the results, we re-used the published estimates of discount factors. The experience of the new group of participants was only different from follow-up group in the temporal proximity of two tasks: intertemporal choice and timing (Figure S2). So, this difference in the dates of the two bi-weekly delay discounting sessions should not play a role for the fitting.

We decided to check whether the fits were robust to re-grouping by comparing (a) the fits from a BHM model just on the follow-up subset of participants (this was done primarily to compare objective to subjective time model fits in the follow-up group) to the fits done earlier in Lukinova et al.[1] in Figure S3A and (b) the fits from a BHM model that merged the follow-up and the new groups to the fits from separate BHM models for each group individually in Figure S3B-C. The new fits were almost identical to the previous fits and, therefore, robust to the re-groupings (Figure S3).

Individual timing

Subjects reported their subjective time through time estimation and time production tasks. Reported or produced values for each trial and the average measures $Te(t)$ and $Tp(t)$ were displayed for each participant in Figure S4 (multi-page PDF is available at github).

To assess whether individual subjects conformed to scalar variability, we fit a linear model of the standard deviation, σ, of subjects' reported intervals (separately for estimation and production) to the actual intervals and computed the r^2, variance explained, by the model for each subject. Since we only had three samples per subject per interval (for each task) estimation of σ for each interval is relatively noisy. Nonetheless, most of our subjects show high values of r^2 in both tasks (Figure S5).

In the main text we reported internal clock correlation results and distributions for subjects altogether. This was justified by the results of the permutation tests, i.e. there was not enough evidence for significant differences between the follow-up and the new group of subjects (*ICSe* as a proxy for ICS: follow-up $M = 0.85$ vs. new $M = 0.90$, permutation test, $p = .346$; *ICSp* as proxy for ICS: follow-up $M = 0.88$ vs. new $M = 0.92$, permutation test, $p = .595$; ICS error: follow-up $M = 0.19$ vs. new $M = 0.18$, permutation test, $p = .770$).

Subjective time estimation

The α (both for power and linear functions) and β were estimated per subject jointly (follow-up and new groups combined) using `brms` package in R[2] according to the equations for the power and linear models, Eq. 6 and Eq. 7 (main text), respectively. Therefore, the power model had two population level parameters and two subject level parameters (α and β), the linear model had one population level parameter and one subject level parameter (α). Both mixed-effects models had suppressed intercepts.

The fits were done using gaussian ('_g' notation in the model name) and exgaussian (according to 'brms' manual *exgaussian*, 'exponentially modified Gaussian' is especially suited to model reaction times) family functions. The best two fits (one power and one linear according to 10-fold cross validation criteria using 'kfold', Table S2) per timing task were then used to substitute delays in the subjective time models, i.e. models that were done taking into account individual subjective time as delay in the short task.

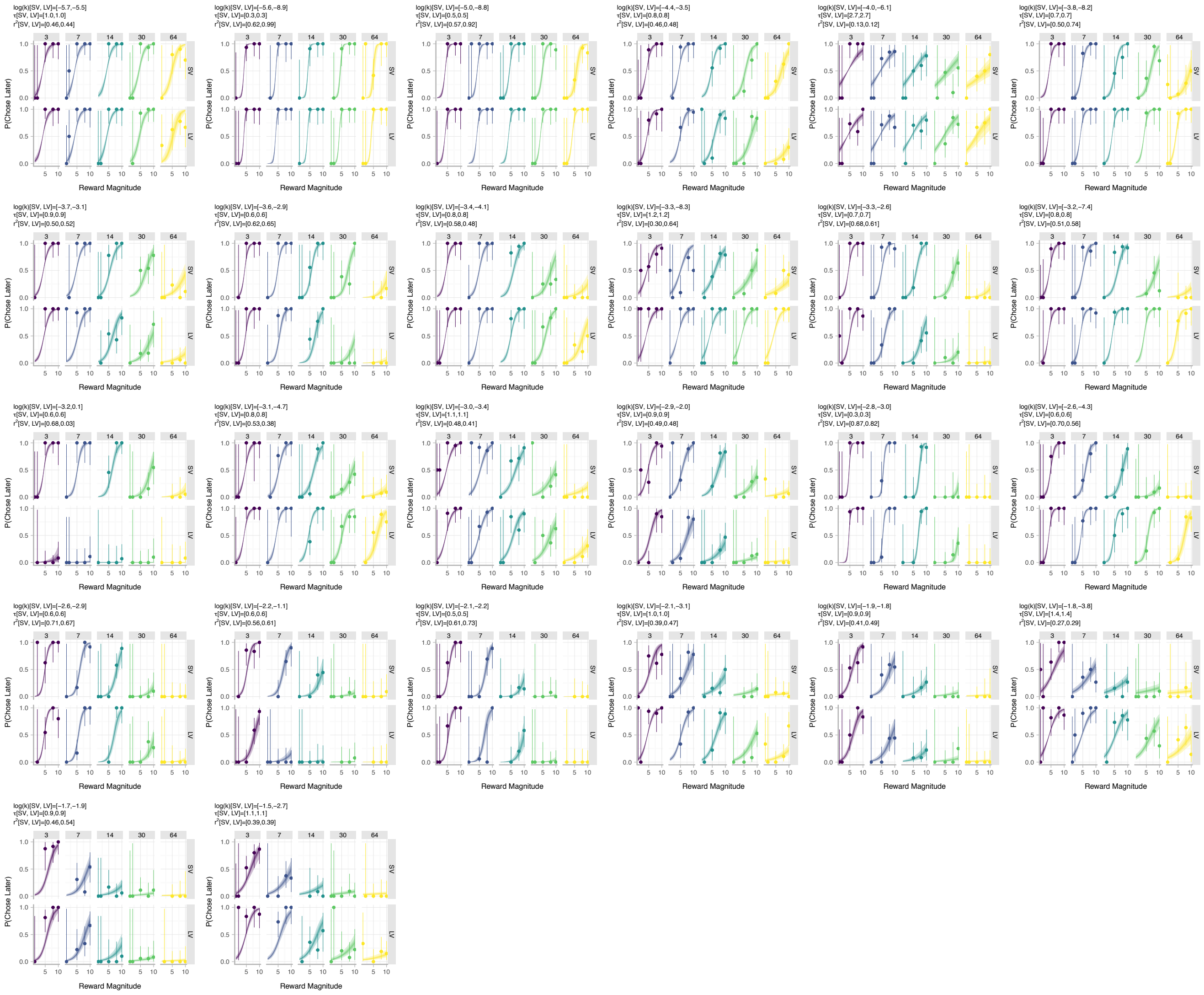

Figure S1. Each plot is the softmax-hyperbolic fit for each subject in the intertemporal choice task (new group, $N = 26$). In each panel, the marker and error bar indicate the mean and binomial confidence intervals of the subjects choices for that offer. The smooth ribbon indicates the BHM model fits (at 50, 80, 99% credible intervals). At the top of each subject plot we indicate the mean estimates of $\log(k)$ and τ for each task for that subject. We also indicate the Bayesian r^2 for each task. Plots from left to right, row-by-row are ordered by discount factor (as estimated using BHM) for the verbal short delay task (SV).

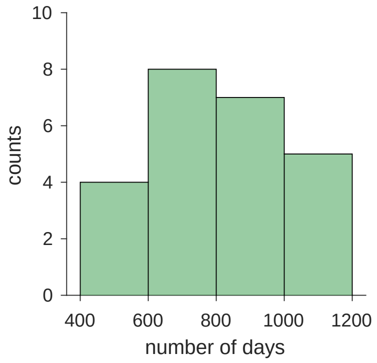

Figure S2. The histogram of time lags between the intertemporal choice task and the timing task: (y-axis) number of subjects as counts; (x-axis) number of days between two experiments.

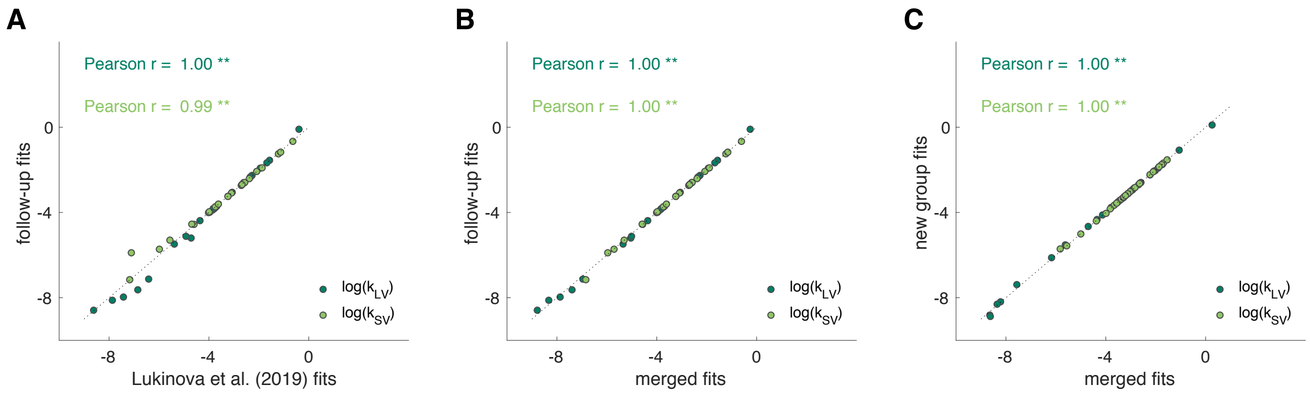

Figure S3. Comparison of discount factors across different model fits. The color of the circles identifies the discount factors for short delay verbal task ($\log(k_{SV}) \sim 1/sec$) or for long delay verbal task ($\log(k_{LV}) \sim 1/day$). Thus, each subject data corresponds to two circles. The dotted line is the unity line $y = x$. Pearson's r is reported on the figure. (A) Follow-up ($N = 24$) discount factors from the main and control experiment 1 in Lukinova et al.[1] (x-axis) are plotted against the separate follow-up fits (y-axis). (B) Follow-up group fits from merged group model (x-axis) plotted against the follow-up fits (y-axis). (C) New group fits from merged group model (x-axis) are plotted against the new group fits ($N = 26$).

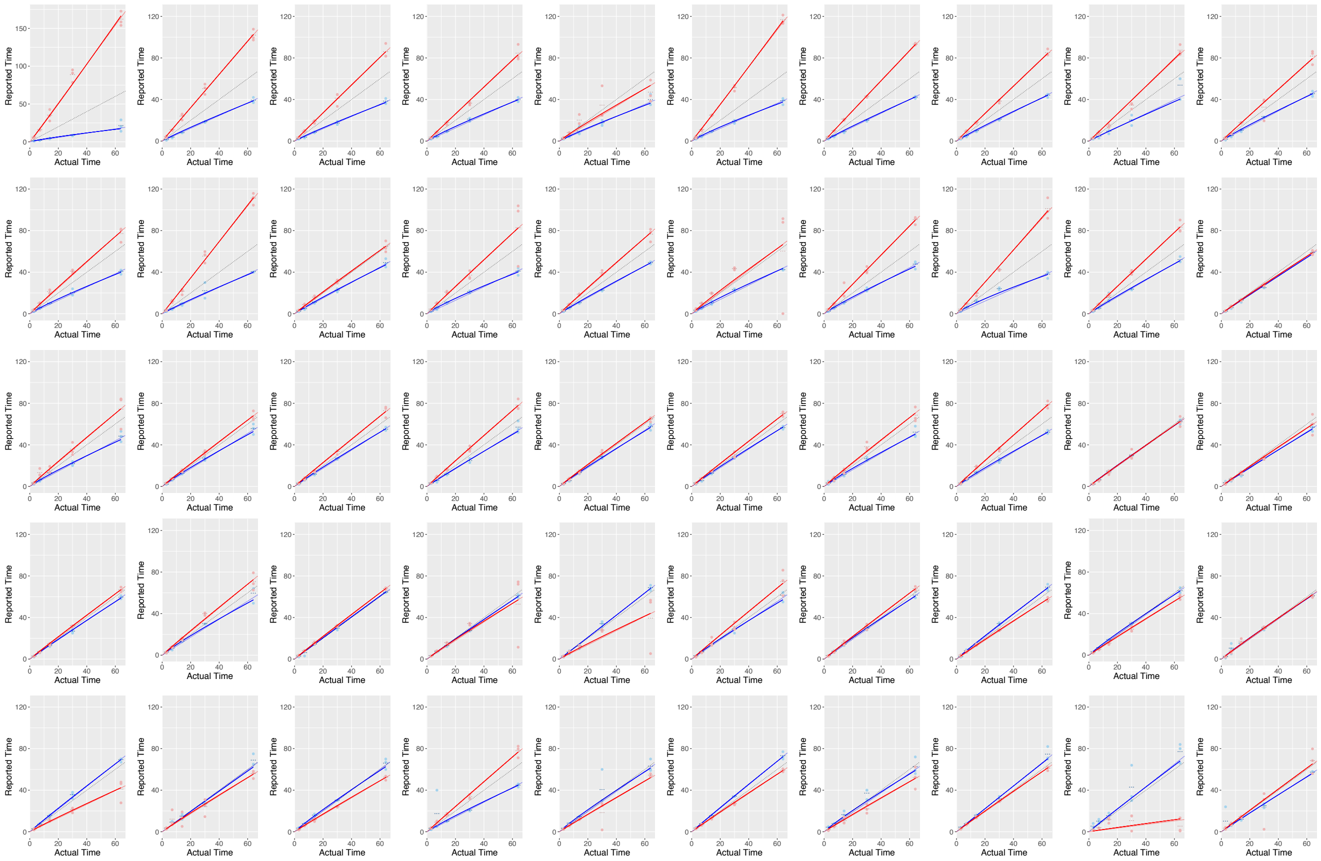

Figure S4. Reported (y-axis) vs. actual (x-axis) time for each subject in the timing tasks. The color aligns with two different timing tasks: time perception (estimation) in blue color palette and time production in red. Circles in each plot correspond to reported time in each trial of the timing experiment, three estimations and three productions per actual time interval. The dash is the calculated average per time interval: $Te(t)$ in dark blue and $Tp(t)$ in dark red for estimation and production, respectively. The dotted line is the unity line $y = x$. The solid and dashed lines of each color are the power and the linear fits, respectively. Plots from left to right, row-by-row are ordered by $ICSe$. Thus, the first plot is the data of the outlier subject, who is removed from analysis whenever proxies for ICS and $ICSError$ were used.

Interval	Production Mean	Production S.D	Estimation Mean	Estimation S.D.
3	3.14309	0.804269	2.68667	1.95979
7	8.11058	2.41703	6.22667	3.29968
14	16.3845	4.72889	12.0267	2.77319
30	34.3773	12.1479	25.8333	7.08284
64	71.5331	25.0484	53.7067	12.1564

Table S1. Table of the mean and standard deviation of reported intervals for the two tasks. This data is represented visually in Figure S5A. n=1500 responses (5 intervals, 3 repeats, 2 tasks, 50 subjects).

	ΔELPD	Δ*SE*
time estimation		
te_power	0.0	0.0
te_linear	-72.0	30.0
te_power_g	-245.1	68.4
te_linear_g	-245.6	70.4
time production		
tp_linear_g	0.0	0.0
tp_power_g	-14.3	13.1
tp_linear	-239.4	105.9
tp_power	-393.1	216.3

Table S2. kfold power and linear model comparison: the 2nd column shows the difference between the expected log pointwise predictive density (ΔELPD[3]) of the best (the first row) model with the model on that row.

Regression analysis

We ran linear regressions according to equations 4 and 5 separately for the followup and the new groups (main text, Methods). The remaining regression analysis tables were displayed in this section.

	Dependent variable: $\log(k_{SV})$	
	(1)	(2)
$\log(k_{LV})$	0.433*	0.398*
	(0.166)	(0.155)
ICSe	3.359	
	(3.359)	
ICSp	-0.690	
	(2.922)	
ICSError	0.977	
	(5.291)	
Constant	-4.132	−1.828*
	(4.315)	(0.720)
Observations	23	23
R^2	.287	.239
Adjusted R^2	.129	.203
Residual Std. Error	1.646 (df = 18)	1.575 (df = 21)
F Statistic	1.811 (df = 4; 18)	6.587** (df = 1; 21)
Note:		*p<.05; **p<.01

Table S3. Follow-up Group $\log(k_{SV})$ Regression Results

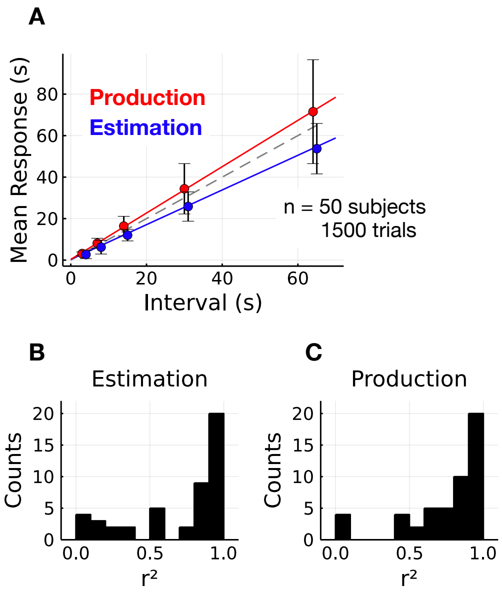

Figure S5. Subjects performance is consistent with scalar timing. A. The mean and standard deviation across all responses for each interval and task. The standard deviation grows with the interval. These data are also presented as Table S1. Production in red and Estimation in blue. The position of the points for Estimation (blue) on the x-axis were shifted to more easily visualize the standard deviation. The dashed line shows the unity line (y=x). The blue and red lines are regression lines for the two tasks. B & C. The two histograms show the range, across subjects, of variance in standard deviation of reported interval explained by a linear model of the actual interval, r^2. High values of r^2 are consistent with scalar timing. The raw data is available at `"https://raw.githubusercontent.com/erlichlab/delayTP/master/data/timing_trials.csv"`

	Dependent variable: $\log(k_{LV})$	
	(1)	(2)
$\log(k_{SV})$	1.555**	1.388**
	(0.348)	(0.349)
ICSe	-4.888	
	(3.189)	
ICSp	0.792	
	(2.665)	
ICSError	−6.551*	
	(3.118)	
Constant	5.725*	0.313
	(2.429)	(1.171)
Observations	26	26
R^2	.556	.397
Adjusted R^2	.471	.372
Residual Std. Error	1.797 (df = 21)	1.958 (df = 24)
F Statistic	6.574** (df = 4; 21)	15.822** (df = 1; 24)
Note:		*p<.05; **p<.01

Table S4. New Group $\log(k_{LV})$ Regression Results

	Dependent variable: $\log(k_{LV})$	
	(1)	(2)
$\log(k_{SV})$	0.631*	0.600*
	(0.243)	(0.234)
ICSe	-4.311	
	(4.042)	
ICSp	2.294	
	(3.492)	
ICSError	1.458	
	(6.386)	
Constant	-0.557	-2.047^{*}
	(5.339)	(0.906)
Observations	23	23
R^2	.310	.239
Adjusted R^2	.157	.203
Residual Std. Error	1.988 (df = 18)	1.933 (df = 21)
F Statistic	2.023 (df = 4; 18)	6.587** (df = 1; 21)
Note:		*p<.05; **p<.01

Table S5. Follow-up Group $\log(k_{LV})$ Regression Results

Demographics and BIS

We used permutation tests to compare timing and discount factors between demographic categories. We did not find any effects of gender or nationality (all $p > .1$) across discount factors ($\log(k_{SV})$, $\log(k_{LV})$) and timing variables ($ICSe$, $ICSp$, $ICSError$).

We used the Barratt Impulsiveness Scale (BIS-11[4]) as a standard measure of impulsivity. The mean total score for the new group was 64.31 (std = 9.57), which was consistent with other reports in the literature[5]. In Lukinova et al.[1] we did not find significant correlations between discount factors and the BIS. We replicated those null results with the new group of subjects: no significant correlations between BIS and (a) $\log(k_{SV})$ (Pearson $r = -.10$, $p = .640$) or (b) $\log(k_{LV})$ (Pearson $r = -.02$, $p = .909$).

Impulsive individuals may be characterized by an impairment in their ability to make temporal judgements. However, studies provide inconsistent and contradictory evidence on the relationship between BIS and timing[6]. In line with Corvi et al.[7], we did not find significant relationship (for the follow-up and the new group separately and combined, the correlation coefficients were reported for the latter) between BIS and timing variables: (a) $ICSe$ (Pearson $r = .11$, $p = .432$), (b) $ICSp$ (Pearson $r = .13$, $p = .375$), (c) $ICSError$ (Pearson $r = -.10$, $p = .482$).

Post hoc analysis

Although this analysis was not preregistered, we checked whether timing was related to decision noise in the intertemporal choice task and performed a joint group analysis.

For the former, we performed correlation analysis between decision noise ($\log(\tau)$) and timing variables in Figure S6A. For the follow-up group, all correlations were not significant: between $\log(\tau)$ and $ICSe$ (Pearson $r = -.00$, $p = .986$), $ICSp$ (Pearson $r = .22$, $p = .316$), $ICSError$ (Pearson $r = -.08$, $p = .705$). For the new group, only the correlation between $\log(\tau)$ and $ICSe$ was significant (Pearson $r = .50$, $p = .009$). Nevertheless, this relationship was mainly driven by a few decision noise outliers and did not hold just for participants with a slower clock ($ICSe < 1$, the majority of our participants, Pearson $r = .05$, $p = .844$ for the new group). The correlation between $\log(\tau)$ and $ICSp$ resulted in Pearson $r = .28$, $p = .167$, between $\log(\tau)$ and $ICSError$ - in Pearson $r = .12$, $p = .552$. The similar pattern was for the joint group analysis: between $\log(\tau)$ and $ICSe$ (Pearson $r = .30$, $p = .034$), $ICSp$ (Pearson $r = .26$, $p = .070$), $ICSError$ (Pearson $r = .00$, $p = .983$).

The joint group analysis was used to test whether the increase in statistical power would change the null results we got when exploring the association of timing with discounting. In the main text we already listed some of the results of the joint analysis. Here in figures S6B-C, we re-plot correlations of timing variables with discount factors separately for the followup and the new groups. As in the joint analysis, there were no significant correlations between ICS measures and delay discounting coefficients. Also, we re-plot the kernel density estimations of $ICSe$ as proxy for ICS divided by differences of subject's impulsivity in seconds compared to that in days in figures S6D-E separately for two groups. The permutation tests confirmed that there were no significant differences in $ICSe$ between $K_{LV} > K_{SV}$ and $K_{SV} > K_{LV}$ subgroups for the follow-up group, $M_{K_{LV}>K_{SV}} = 0.87$

and $M_{K_{SV}>K_{LV}} = 0.84$, $p = .624$ and for the new group, $M_{K_{LV}>K_{SV}} = 0.83$ and $M_{K_{SV}>K_{LV}} = 0.94$, $p = .178$. Thus, as in the joint analysis in the main text, we did not find any significant difference in internal clocks between subjects who were more impulsive in the seconds task than the days task and those, who were more impulsive in the days task than the seconds task.

In the joint analysis (compared to the new group analysis in the main text), dropping *ICSe* (a proxy for ICS) no longer resulted in a significant decrease in the likelihood for explaining short delay in Figure S6F. As before, we generated linear regression models of $\log(k)$ for each task (short delay and long delay) against the discount factor of the other task, as well as timing variables. In order to test which factors were important, we dropped each factor and tested whether the decrease in likelihood was significant by a χ^2 test. We plot the change in AIC, with significant drops in black ($p < .0125$, Bonferroni Corrected $p < .05/4$).

Further, we looked at the possibility of a delay-dependent relationship of timing with discounting. According to Figure S6G, there was no evidence that people who tend to overestimate 64 seconds (or other relatively longer delays) were more impulsive or more patient for 64 second delays (or other relatively longer delays) compared to the relatively smaller delays that were estimated or produced more accurately.

In addition, we used the following linear models to test the contribution of timing variables to each discount factor while controlling for the group the subjects belonged to:

$$\log(K_{SV}) \sim ICSe + ICSp + ICSError + \log(K_{LV}) + group \tag{1}$$

$$\log(K_{LV}) \sim ICSe + ICSp + ICSError + \log(K_{SV}) + group \tag{2}$$

The detailed regression results in Tables S6 and S7 for joint groups data were similar to the respective regression tables here and in the main text. There was no main effect of the group. In the reduced models, we found that $\log(k_{LV})$ significantly predicted $\log(k_{SV})$ (Table S6) and $\log(k_{SV})$ significantly predicted $\log(k_{LV})$ (Table S7). These reduced models contained the only factors that mattered (according to the joint 'drop 1' analysis in Figure S6F). As in the main text, we also considered the model where two regressors ($\log(k_{LV})$ and *ICSe*) predict $\log(k_{SV})$ in Table S6. With the addition of *ICSe* we explained 34% (an increase in 5% compared to 29% for reduced model) variance, but the coefficient for *ICSe* was not significant.

	Dependent variable: $\log(k_{SV})$		
	(1)	(2)	(3)
$\log(k_{LV})$	0.359**	0.331**	0.350**
	(0.078)	(0.078)	(0.077)
ICSe	3.105*		1.776
	(1.492)		(0.971)
ICSp	-0.851		
	(1.235)		
ICSError	1.443		
	(1.684)		
group	0.174	0.287	0.195
	(0.354)	(0.358)	(0.352)
Constant	−4.166**	−2.104**	−3.543**
	(1.197)	(0.415)	(0.884)
Observations	49	49	49
R^2	.365	.289	.338
Adjusted R^2	.291	.258	.294
Residual Std. Error	1.222 (df = 43)	1.250 (df = 46)	1.219 (df = 45)
F Statistic	4.939** (df = 5; 43)	9.344** (df = 2; 46)	7.663** (df = 3; 45)
Note:			*p<0.05; **p<0.01

Table S6. Joint Group $\log(k_{SV})$ Regression Results

Using subjective time, rather than objective time in joint analysis did not improve our ability to predict subjects' choices (Table S8). In fact, when we used subjective time from time production task we significantly decreased the correlation between the delay discounting coefficients.

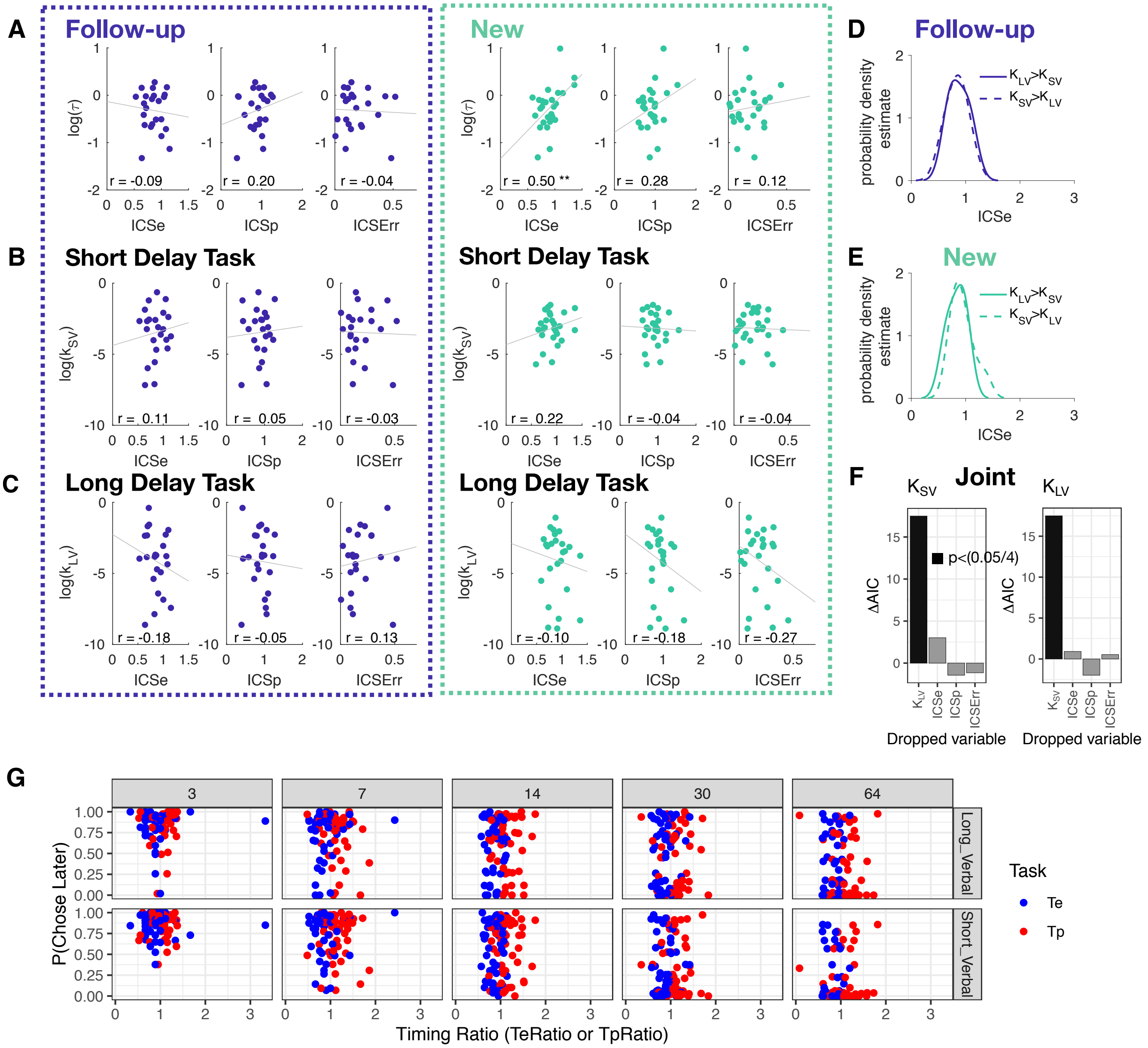

Figure S6. (A-C) Correlation plots ($N = 49$, separately for the followup and new groups arranged in columns highlighted by a colored border) between noise and discount factors (y-axis, rows) and $ICSe$, $ICSp$, and $ICSError$ (x-axis). Each circle is one subject. Pearson's r is reported on the figure (** $p < .01$; for all correlations with discount factors $p > .05$). Best linear fit line ($y \sim x$) is displayed. (D-E) Kernel density estimations of $ICSe$ and $ICSp$ as proxies for ICS and $ICSError$ divided by positive or negative difference between discount factor in short and long delay tasks for both of our subjects' groups. (F) Drop-one regression analysis for joint data no longer resulted in a significant decrease in the likelihood for any variables dropped, except for the discount factor. (G) Relationship between timing ratio ($TeRatio(t)$ and $TpRatio(t)$, x-axis) and the proportion of later choices (y-axis) grouped by a particular delay (or actual time interval, columns), intertemporal (rows) and timing (color) tasks.

	Dependent variable: $\log(k_{LV})$	
	(1)	(2)
$\log(k_{SV})$	0.912**	0.848**
	(0.199)	(0.200)
ICSe	-3.885	
	(2.423)	
ICSp	-0.252	
	(1.978)	
ICSError	-3.981	
	(2.636)	
group	-0.068	-0.213
	(0.565)	(0.575)
Constant	3.337	-1.185
	(2.098)	(0.810)
Observations	49	49
R^2	.363	.281
Adjusted R^2	.289	.250
Residual Std. Error	1.946 (df = 43)	1.999 (df = 46)
F Statistic	4.904** (df = 5; 43)	8.993** (df = 2; 46)
Note:		*p<.05; **p<.01

Table S7. Joint Group $\log(k_{LV})$ Regression Results

	Pearson *r* value	*p* value	Significantly Better?
obj	.51	< .001	
subjTel	.54	< .001	no
subjTep	.54	< .001	no
subjTpl	.43	.002	no
subjTpp	.44	.001	no

Table S8. Pearson Correlation between $\log(k_{SV})$ and $\log(k_{LV})$. 'Significantly Better?' answers whether there was significant increase in correlations from the objective time model to the respective subjective time model tested using R package `cocor`.

References

1. Lukinova, E., Wang, Y., Lehrer, S. F. & Erlich, J. C. Time preferences are reliable across time- horizons and verbal versus experiential tasks. *eLife* 27, DOI: https://doi.org/10.7554/eLife.39656.001 (2019).

2. Bürkner, P.-C. brms: An R Package for Bayesian Multilevel Models Using Stan. *J. Stat. Softw.* **80**, 1–28, DOI: 10.18637/jss.v080.i01 (2017).

3. Vehtari, A., Gelman, A. & Gabry, J. Practical bayesian model evaluation using leave-one-out cross-validation and waic. *Stat. Comput.* **27**, 1413–1432 (2017).

4. Patton, J. H., Stanford, M. S. & Barratt, E. S. Factor structure of the barratt impulsiveness scale. *J. clinical psychology* **51**, 768–774 (1995).

5. Stanford, M. S. *et al.* Fifty years of the barratt impulsiveness scale: An update and review. *Pers. individual differences* **47**, 385–395 (2009).

6. van den Broek, M., Bradshaw, C. & Szabadi, E. Performance of impulsive and non-impulsive subjects on two temporal differentiation tasks. *Pers. Individ. Differ.* **13**, 169–174, DOI: 10.1016/0191-8869(92)90039-R (1992).

7. Corvi, A. P., Juergensen, J., Weaver, J. S. & Demaree, H. A. Subjective time perception and behavioral activation system strength predict delay of gratification ability. *Motiv. Emot.* **36**, 483–490, DOI: 10.1007/s11031-011-9275-0 (2012). Tex.ids: corviSubjectiveTimePerception2012a.
